# Insights into potential pathogenesis mechanisms associated with *Campylobacter jejuni*-induced abortion in ewes

**DOI:** 10.1186/s12917-014-0274-8

**Published:** 2014-11-25

**Authors:** Yasser M Sanad, Kwonil Jung, Isaac Kashoma, Xiaoli Zhang, Issmat I Kassem, Yehia M Saif, Gireesh Rajashekara

**Affiliations:** Food Animal Health Research Program (FAHRP), Ohio Agricultural Research and Development Center, 1680 Madison Avenue, Wooster, OH 44691 USA; Center for Biostatistics, The Ohio State University, Columbus, OH 43220 USA; Veterinary Preventive Medicine Department, The Ohio State University, 1680 Madison Avenue, Columbus, OH 43220 USA; Current address: Division of Microbiology, National Center for Toxiocological Research, US Food and Drug Administration, Jefferson, USA

**Keywords:** *Campylobacter jejuni*, Sheep abortion, Pathogenesis, Public health, Pregnant ewes

## Abstract

**Background:**

*Campylobacter jejuni* is commonly found in the gastrointestinal tract of many food-animals including sheep without causing visible clinical symptoms of disease. However, *C. jejuni* has been implicated in ovine abortion cases worldwide. Specifically, in the USA, the *C. jejuni* sheep abortion (SA) clone has been increasingly associated with sheep abortion. I*n vivo* studies in sheep (the natural host) are needed to better characterize the virulence potential and pathogenesis of this clone.

**Results:**

Pregnant ewes intravenously (IV) or orally inoculated with ovine or bovine abortion-associated *C. jejuni* SA clones exhibited partial or complete uterine prolapse with retained placenta, and abortion or stillbirth, whereas delivery of healthy lambs occurred in pregnant ewes inoculated with *C. jejuni* 81–176 or in the uninfected group. In sheep inoculated with the SA clone, histopathological lesions including suppurative necrotizing placentitis and/or endometritis coincided with: 1) increased apoptotic death of trophoblasts, 2) increased expression of the host genes (e.g. genes encoding interleukin IL-6 and IL-15) related to cellular necrosis and pro-inflammatory responses in uterus, and 3) decreased expression of the genes encoding GATA binding protein 6, chordin, and insulin-like 3 (INSL3) that account for embryonic development in uterus. Immunohistochemistry revealed localization of bacterial antigens in trophoblasts lining the chorioallantoic membrane of ewes inoculated with the *C. jejuni* SA clone.

**Conclusions:**

The results showed that *C. jejuni* SA clones are capable of causing abortion or stillbirth in experimentally infected sheep. Furthermore, down- or up-regulation of specific genes in the uterus of infected pregnant ewes might implicate host genes in facilitating the disease progression. Since the *C. jejuni* SA strains share genotypic similarities with clones that have been isolated from human clinical cases of gastroenteritis, these strains might represent a potential public health risk.

**Electronic supplementary material:**

The online version of this article (doi:10.1186/s12917-014-0274-8) contains supplementary material, which is available to authorized users.

## Background

*Campylobacter jejuni* is a leading cause of bacterial gastroenteritis in humans, resulting in diarrhea and abdominal pain. *C. jejuni* is commonly found in the intestinal tract of many food-producing animals, such as poultry, cattle, and sheep [1]. In these hosts, *C. jejuni* generally persists without causing visible clinical symptoms of disease. Although it has been historically known that *C. fetus subsp fetus* (vibriosis) is the main cause of ovine abortion, recently *C. jejuni* has been increasingly implicated in ovine abortion cases worldwide [2-5]. *Campylobacter*-induced ovine abortion rates usually range from 5% to 50% in infected flocks [4,6]. *C. jejuni* has been identified in aborted lambs during multiple lambing seasons on different farms in Iowa, Idaho, South Dakota, and California [5]. A specific tetracycline-resistant *C. jejuni* clone (SA) has emerged as the major cause of *C. jejuni*-associated sheep abortion in the USA [5]. In addition, clone SA has been also detected in abortion cases in cows and goats [7].

The pathogenesis of ovine campylobacteriosis is thought to include bacteremia, placentitis and uterine and fetal infection, which may ultimately lead to abortion that typically occurs in the third trimester of pregnancy [4,8]. Furthermore, infection can occasionally result in the retention of dead fetus in the uterus which may cause death of pregnant ewes due to septicemia and uterine sepsis; however, no clinical signs can be seen at the beginning of infection [4]. The mechanisms by which the gut commensal *C. jejuni* is able to establish systemic infection and lead to an acute abortion in sheep are still not completely defined. Furthermore, in a recent study, PFGE analysis of *C. jejuni* SA isolates revealed a high similarity to human gastroenteritis-associated *C. jejuni* isolates [7]. This suggested a potential relevance of these ovine abortion isolates to human health. Therefore, there is a critical need for understanding the pathogenesis of *C. jejuni* induced abortion.

Using guinea pigs, abortion was effectively induced in pregnant animals intra-peritoneally or orally inoculated with *C. jejuni* clone SA, and the bacteria were recovered from fetuses and placental tissues [9]. These findings have indicated that ovine abortion-associated *C. jejuni* is highly abortificient and guinea pigs are a useful model to understand the pathogenesis of abortion. However, further *in vivo* studies in a natural host (sheep) are needed to better characterize the virulence potential and pathogenesis of the abortion-associated *C. jejuni*. These studies would aid in the development of effective control or prevention strategies.

In order to gain more insights into the pathogenesis of *C. jejuni* induced abortion in sheep, *in vivo* pathogenesis studies of ovine abortion were conducted using pregnant ewes and host gene expression profiles were compared between infected and uninfected ewes using microarray analysis. Overall, the results suggested that bovine and ovine *C. jejuni* clone SA can induce abortion in pregnant ewes by causing necrotizing suppurative placentitis and endometritis, which was also supported by increased expressions of host genes related to cellular necrosis and suppurative inflammation (pro-inflammatory cytokines).

## Methods

### Ethics statement

Animal experiments were conducted according to the guidelines of the Association for Assessment and Accreditation of Laboratory Animal Care International (AAALAC). The animal studies were approved by the Agricultural Animal Care and Use Committee (AgACUC), OARDC, The Ohio State University (OSU) under the protocol number 2011A00000028. By necessity, microbial pathogenesis studies are heavily focused on the use of *in vivo* models, because the host immune responses interplay with pathogen evasion constitute a complex response that cannot yet be accurately replicated *in vitro*. Experiments were designed to use the minimal number of animals (4 ewes per group) necessary to generate interpretable data. Specifically, based on the *C. jejuni* associated abortion rates reported in the literature (50%) and preliminary studies, using 4 animals per group yielded a power of 0.85 (α =0.05). Since the objective of this study was to understand the complex host-pathogen interactions, the use of laboratory animals was unavoidable and justifiable.

Pregnant ewes were housed at the Food Animal Health Research Program Animal Care Facility which is fully accredited by AAALAC. Infectious agents were administered using manual restraint for less than one minute to minimize distress. Sheep were euthanized using phenobarbital injection i/v consistent with the recommendations of the panel on euthanasia of the American Veterinary Medical Association and by the Ohio State University Institutional Laboratory Animal Care and Use Committee.

### Abortion associated *C. jejuni* isolates

Three *Campylobacter* isolates were obtained from the Ohio Department of Agriculture (ODA, Columbus, OH). Two *C. jejuni* isolates (Ovine abortion-I and Ovine abortion-II) were retrieved from aborted Suffolk sheep fetuses from ewes in their third trimester of pregnancy in 2008, and one *C. jejuni* isolate (Bovine abortion) was isolated from an aborted cow in 2009. These isolates (Ovine abortion-I, Ovine abortion-II, and Bovine abortion) were confirmed as *C. jejuni* using a genus as well as a species specific PCR [10,11]. Furthermore, PFGE analysis [11-13] showed that the ovine and bovine abortion isolates possessed identical profiles. In addition, MLST analysis confirmed that these abortion-associated *C. jejuni* belonged to the same previously described abortion associated tetracycline resistant clone SA [sequence type (ST) 8; clonal complex (CC) 21] [5,11,13,14] (Additional file 1: Figure S1).

### Inoculation of pregnant ewes with the ovine and bovine abortion *C. jejuni* isolates

Four groups of ewes in the third trimester of pregnancy were used. These ewes were obtained from the OSU sheep facility herd which had no history of abortions and was vaccinated only against *Chlamydia* and *Toxoplasma*. Ewes were tested for pregnancy with ultrasound at approximately 8–9 weeks after insemination and then pregnant ewes (14–15 weeks) were inoculated with *C. jejuni* strains. Fecal samples were collected from all ewes one week before inoculation and examined for the presence of *Campylobacter*. Each inoculated group consisted of four ewes. The first group was inoculated with a common laboratory strain, *C. jejuni* 81–176, (G1). The second and third groups were inoculated with (Bovine abortion: G2) and (Ovine abortion-II: G3) isolates, respectively. In each group, two ewes were inoculated with *C. jejuni* via oral route and the other two were inoculated i/v. Oral inoculation simulated the natural route of *C. jejuni* infection in sheep; however, to insure that the infection will occur, sheep were also inoculated i/v. The fourth group, which consisted of two ewes, was used as a non-inoculated control. Ewes were either drench inoculated orally with 50 ml sterile PBS containing 10^10^ CFU ml^-1^ or via i/v using a syringe with 1.5 ml sterile PBS containing 10^9^ CFU. These doses were selected based on previous studies [8,15]. The ewes were monitored after infection until abortion or the end of pregnancy.

### Necropsy of ewes and bacteriological examination

Ewes and fetuses were necropsied immediately following abortion or normal delivery. Maternal tissues: caruncles and placenta, liver, kidney, spleen, lungs, uterus, small intestine, blood, colostrum, placental fluids, and feces; and fetal tissues: small intestine, lung, liver, meconium, cecal contents, fetal fluids (abdominal fluids and blood) were collected and processed for microbiological examination individually. The tissues were aseptically collected using separate sterilized sets of necropsy tools for each organ. To detect *C. jejuni*, 1 g of each tissue was enriched in Preston broth for 48 h at 42°C under microaerobic conditions. An (100 μl) inoculum from the enrichments was spread onto modified Cefoperazone Charcoal Deoxycholate Agar (mCCDA) plates, which were then incubated for an additional 48 h at 42°C under microaerobic conditions [16]. Furthermore, an inoculum (100 μl) from placental and uterine tissues of each animal was plated directly onto mCCDA plates, respectively. The plates were then incubated as described previously and the CFU were counted. The growth was categorized as + = (mild growth: less than 100 CFU), ++ = (moderate growth: less than 300 CFU), +++ = (heavy growth: too many to count), -- = (no growth).

### Histopathological studies

A histopathological examination [17] was conducted to determine the pathological changes in infected and control ewes and fetal tissues. Samples collected from the intestine, liver, kidney, spleen, placenta, and uterus as well as tissue samples collected from lambs (lung and small intestine) after normal delivery or from aborted and/or stillbirth fetuses were examined for histological changes. Tissues were placed in 10% phosphate buffered formaldehyde (pH 7.0), dehydrated in graded alcohol, embedded in paraffin, and cut in 3-μm sections onto microscope slides. The slides were fixed and stained with hematoxylin and eosin (H&E) and samples were blinded prior to analysis for histopathological changes by a pathologist.

### Immunohistochemistry (IHC)

Monoclonal mouse anti-*C. jejuni* antibody clone B082M (Acris Antibodies, Inc., San Diego, CA) was used for the detection of *C. jejuni* antigens in formalin-fixed, paraffin-embedded tissues. The antibody was diluted 1 in 200 in PBST [phosphate-buffered saline (PBS) containing 0.1% Tween 20]. IHC was carried out using an in house protocol that was developed for pig tissues as described previously [17]. Paraffin-embedded tissues were sectioned at 3 μm thickness and collected on positively charged slides (Fisher Scientific, PA). The slides were kept at 60°C for 20 min, deparaffinized in xylene twice for 5 min each, and rehydrated through a graded ethanol series (100% to 50%). Endogenous alkaline phosphatase was quenched with glacial acetic acid (20%) for 2 min at 4°C. Antigen retrieval was performed using 100 μg ml^-1^ of proteinase K (Invitrogen, Carlsbad, CA). The tissue slides were then washed in PBST three times and blocked with 1× buffered solution of casein (Universal Blocking Reagent; Biogenex, Fremont, CA) in distilled water for 30 min at room temperature to saturate nonspecific protein-binding sites. Sections were coated with monoclonal antibodies and incubated overnight at 4°C in a humid chamber. After three washes with PBST, the sections were flooded and incubated for 1 h at 37°C with goat anti-mouse IgG labeled with alkaline phosphatase (Dako, Glostrup, Denmark) diluted 1 in 200 in PBST. After three washes with PBST, the final reaction was generated by immersing the tissue sections in a staining solution [1 tablet of Fast Red in 2 ml of 0.1 M Tris-buffer (pH 8.2); Roche Applied Science, Mannheim, Germany] for 10 min at room temperature. Sections were lightly counterstained with Mayer’s hematoxylin.

### In situ terminal deoxynucleotidyl transferase-mediated dUTP nick end labelling (TUNEL) assay

Paraffin-embedded placental tissues were prepared as described above and evaluated by an *in situ* TUNEL assay kit (Roche Applied Science) for apoptosis [18] according to the manufacturer’s instructions. The severity of apoptosis was qualitatively estimated based on the distribution and number of *in situ* TUNEL-positive cells in the placenta per microscopic area at × 200 magnification. TUNEL-positive cells (red staining) were then randomly compared between infected and negative control animals.

### Total RNA extraction from uterus

RNA was extracted from uterine tissues of two animals from each group of ewes that undergone abortion or delivered a stillbirth. Similarly, uterine tissues from two control non-inoculated and two 81–176 inoculated sheep were used for RNA extraction. Uterus samples were collected aseptically during necropsy and immediately flash frozen in liquid nitrogen. The samples were then immediately transferred to - 70°C until further analysis. At the completion of the experiment, RNA extraction from the frozen tissues was performed using Qiagen RNeasy® Mini Columns following the manufacturer protocol. Briefly, 30 mg of tissue was lysed in 1 ml TRIzol (Invitrogen) with homogenization (Cole-Parmer, lab Gen 125). Chloroform (0.2 ml) was added, the samples were mixed vigorously and incubated at room temperature for 2–3 min. Samples were centrifuged at 12,000 × g at 4°C for 5 min. The aqueous phase was separated and used for RNA extraction using Qiagen RNeasy® Mini Columns. The RNA was stored at -70°C until further use.

### Microarray analysis

The RNA concentration was determined using the NanoDrop spectrophotometer (NanoDrop products, Wilmington, DE), and the integrity of the RNA was confirmed by gel electrophoresis as well as by using an Agilent 2100 bioanalyzer (Agilent Technologies, Santa Clara CA). The samples with ratio closer to 2 were used for microarray analyses. Microarray expression analysis was performed using *Ovis aries* Sureprint 8 × 15K arrays (Agilent Technologies, Santa Clara CA). The Sureprint sheep array has 60mer oligos representing 15,208 probes.

Microarray hybridizations were performed according to manufacturer’s instructions at the Research Institute at Nationwide Children’s Hospital, Biomedical Genomics Core Facility, (Columbus, OH). In brief, samples were labeled with Cy3, purified using Qiagen columns, and checked for labeling efficiency using the Nanodrop. The labeled samples were fragmented and hybridized to the array overnight. Microarray slides were then washed and scanned with Agilent G2505C Microarray Scanner at 2 μM resolution. Images were analyzed with Feature Extraction 10.10 (Agilent Technologies, CA).

For analysis, the intensity data were background corrected and summarized with quantile normalization method [19,20]. A filtering method was applied if more than 80% of arrays showed that the expression levels of a gene were at or below the noise cutoff. The data from 2 samples were averaged for further analysis. Ingenuity Pathway Analysis (IPA; http://www.ingenuity.com) was used to identify signaling pathways and networks involving genes of interest.

### Statistical analysis

Data describing *C. jejuni* counts and *in vitro* assays were analyzed using one-way ANOVA followed by Tukey’s Multiple Comparison adjustment. A value of *P* < 0.05 was considered statistically significant for all experiments. Measurements were expressed as mean ± SE (standard error) and represent averages of three replicates. For microarray data analysis, linear regression models were used for comparisons among different treatment groups. Due to the small sample size per treatment group, the *P*-values were not adjusted for multiple gene testing within each treatment group comparisons. However, the overall type I error at 0.05 for the multiple group comparisons (4 treatment groups and 6 comparisons) was strongly controlled. Therefore, based on the Bonferroni correction for multiple comparisons test [21], a *P*-value =0.05/6 = 0.008 was used as cutoff to determine if a gene was differentially expressed within a comparison group. Subsequently, the tests were ordered by fold change and *P*-values. As a 2-fold difference is a widely accepted cutoff for biological differences, genes with at least a 2-fold change and a *P*-value < 0.008 were considered differentially expressed.

## Results

### Clinical observations of pregnant ewes after infection

**Figure 1:**
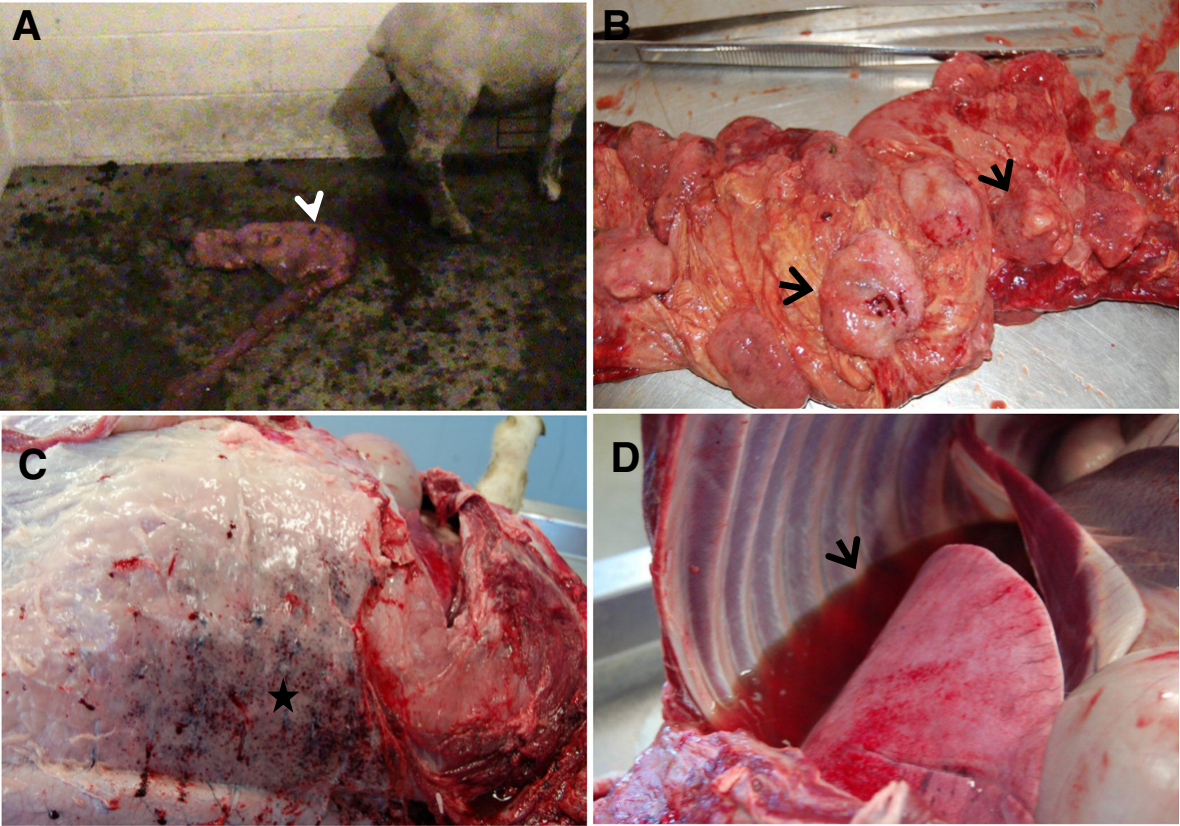
**Gross pathology in ewes inoculated with abortion-associated** ***C. jejuni*** **. A**. Aborted fetus (arrow) of a pregnant ewe after oral inoculation of ovine *C. jejuni* strain*.* **B**. Placenta of the aborted ewe, showing diffuse, severe suppurative placentitis characterized by edematous, hemorrhagic, and fibrinopurulent placentomes (arrows). **C**. Hypodermis of a dead ewe inoculated intravenously with ovine abortion *C. jejuni* isolate, showing multifocal-coalescing petechial hemorrhage (star). **D**. Pleural cavity of a dead ewe inoculated intravenously with ovine abortion *C. jejuni* isolate, showing severe hydrothorax (arrow).

The ovine and bovine abortion-associated *C. jejuni* strains induced abortion or stillbirth. One of the ewes orally inoculated with the ovine isolate (G3-O-2) aborted at PID 15 (Figure 1A). A month later, another ewe (G3-O-1) from the same group delivered a dead fetus. Furthermore, one ewe (G3-V-1) i/v inoculated with the ovine isolate died 20 h after inoculation (Table 1). Rectal temperatures of inoculated ewes were elevated (105–105.5°F) 3 to 4 days post i/v inoculation and at PIDs 10-12 in orally inoculated ewes. Stillbirths were observed in 2 cases in G2 (G2-V-1 and G2-V-2), which were inoculated i/v with the bovine isolate (Table 1), whereas the two ewes orally inoculated in G2 group delivered normally. No clinical signs were observed in the G1 ewes orally or i/v inoculated with 81–176 strain, and they delivered normally. Feces changed from pasty to diarrheic in most of the inoculated ewes, which was profoundly seen in the G2 and G3 groups. No abortions or clinical signs were observed in control non-inoculated ewes (Table 1).

**Table 1:** **Summary of clinical observations and bacteriological studies**

**Groups**	**Control**		**Group1 (G1)**				**Group2 (G2)**				**Group3 (G3)**			
**Animals no.**	**1**	**2**	**G1-O-1**	**G1-O-2**	**G1-V-1**	**G1-V-2**	**G2-O-1**	**G2-O-2**	**G2-V-1**	**G2-V-2**	**G3-O-1**	**G3-O-2**	**G3-V-1**	**G3-V-2**
**Strain used for inoculation**	**None (sterile saline)**		**81–176**				**Bovine abortion** ***C. jejuni***				**Ovine abortion** ***C. jejuni***			
**Status**	Normal delivery	Normal delivery	Normal delivery	Normal delivery	Normal delivery	Normal delivery	Delivered twins	Normal delivery	**Stillbirth**	**Stillbirth**	**Stillbirth**	**Abortion**	**Died 20 h pi**	Normal^a^ delivery
**Occurrence of abortion or delivery post inoculation**	6 weeks	7 weeks	6 weeks	6 weeks	4 weeks	7 weeks	5 weeks	7 weeks	4 weeks	3 weeks	6 weeks	2 weeks	----	6 weeks
**Liver**	--	--	--	--	**+++**	**+**	--	--	**+++**	**+++**	--	**+++**	**+**	--
**Spleen**	--	--	--	--	--	--	--	--	**+**	**+**	--	**+++**	**+**	--
**Kidney**	--	--	--	--	--	--	--	--	--	--	--	**++**	**+**	--
**Blood**	--	--	--	--	--	--	--	--	--	--	--	**+**	**+**	--
**S. Intestine**	--	--	**+**	**+**	**+**	**+**	**+**	**+++**	**++**	**+++**	**+++**	**++**	-- (highly congested)	--
**Feces**	--	--	**+**	**+**	**+**	**+**	**+**	**+**	**+**	**+**	+	**+**	--	--
**Lung**	--	--	--	--	--	--	--	--	--	--	--	**+++**	**+** (hydrothorax)	--
**Placenta**	--	--	--	--	**+**	--	**+**	--	1 × 10^7^	4 × 10^7^	2 × 10^6^	2 × 10^8^	--	--
**Uterus**	--	--	--	--	**+**	--	**+**	**+**	5 × 10^6^	1 × 10^7^	4 × 10^5^	3 × 10^7^	--	--
**Colostrum**	--	--	--	--	**+**	--	--	--	--	--	**+**	**+++**	N/A	--
**Placental fluids**	--	--	--	--	--	--	--	--	**+++**	**+++**	**+**	**+++**	--	--
**Fetal fluids**	--	--	--	--	--	--	--	--	**+++**	**+++**	**+**	**+++**	--	--
**Fetal Liver**	--	--	--	--	--	--	--	--	**+++**	**+++**	--	**+**	N/A	--
**Fetal Lung**	--	--	--	--	--	--	--	--	**+++**	**+++**	--	**+**	N/A	--
**Fetal S. Intestine**	--	--	--	--	--	--	**+**	--	**+++**	**+++**	**+++**	**+**	N/A	--
**Fetal meconium**	--	--	--	--	--	--	**--**	--	**+**	**+**	**+**	**--**	N/A	--

+ = (mild growth: less than 100 CFU), ++ = (moderate growth: less than 300 CFU), +++ = (heavy growth: too many to count), -- = (No Growth), N/A, Not applicable; ^a^Ewe (G3-V-2) no *C. jejuni* was isolated from any tissues; however, at postmortem this ewe was found to be heavily infested with tapeworm.

### Pathological studies

Major macroscopic lesions of abortion-associated *C. jejuni*-inoculated ewes included placentitis and endometritis, regardless of the ovine or bovine strains used. The affected placentomes and uterus appeared edematous, hemorrhagic, and fibrinopurulent (Figure 1B). Enlarged placentomes were more prominent in the G3 ewes orally or i/v infected with the ovine abortion-II isolate. Liver and spleen also exhibited petechial hemorrhages on their surface in ewes that were i/v inoculated with the bovine abortion strain (G2-V-1) as well as in ewes orally or i/v inoculated with the ovine abortion strain (G3-V-1; G3-O-1; G3-O-2). Additionally, hydrothorax and subcutaneous hemorrhage as potential signs of acute septicemia were detected in the dead ewe at 20–24 h after i/v inoculation (Figure 1C and D); however, pneumonia was not accompanied with the former lesion. No gross lesions were noted in G1 or the control ewes.

**Figure 2:**
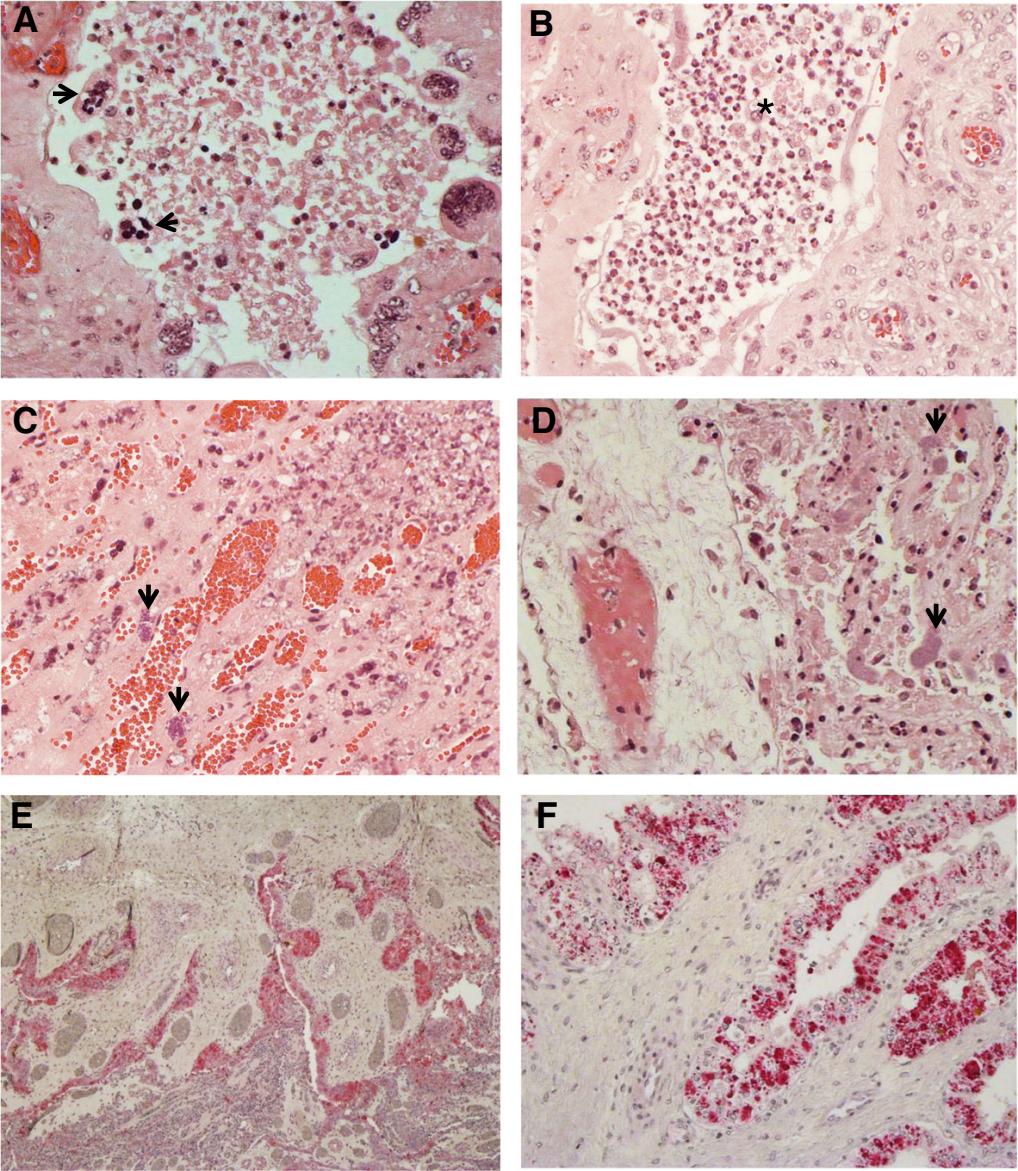
**Pathological findings in placenta of aborted ewe. (A)** Placentome of an aborted ewe experimentally infected orally with ovine *C. jejuni*, showing severe necrotizing placentitis. Note necrotic trophoblast giant cells (arrows) exfoliated from chorionic villi. Original magnification, ×400. **(B)** Placentome of an aborted ewe experimentally infected with ovine *C. jejuni*, showing severe suppurative, necrotizing placentitis. Note a large accumulation of neutrophils and necrotic cells (asterisk) in the intervillous space. Original magnification, ×400. **(C)** Placentome of an aborted ewe experimentally infected with ovine *C. jejuni*, showing bacterial colonies (arrows) within the necrotic lesion. Original magnification, ×400. **(D)** Chorioallantoic membrane of an aborted ewe experimentally infected with ovine *C. jejuni*, showing severe necrosis of infected epithelium and containing bacterial colonies (arrows). Original magnification × 400. **(E)** Placenta of an aborted ewe experimentally infected with ovine *C. jejuni*, showing localization of majority of bacterial antigens (red-color staining) in chorioallantoic membrane. Immunohistochemistry. Fast Red. Mayer’s hematoxylin counterstaining. Original magnification, ×60. **(F)** Chorioallantoic membrane of an aborted ewe experimentally infected with ovine *C. jejuni*, showing large amounts of bacterial antigens in trophoblastic epithelial cells (red-color staining) lining the epithelium. Immunohistochemistry. Fast Red. Mayer’s hematoxylin counterstaining. Original magnification, ×400.

In line with macroscopic observations, histological lesions mainly consisted of suppurative placentitis or endometritis or both. Placentitis was commonly observed widespread throughout placenta, comprising placentomes and chorioallantoic membranes. In the placentomes of inoculated ewes (G2 and G3), trophoblasts lining the placental villi were necrotic with extensive neutrophilic infiltration (Figure 2A and B). Occasionally, small bacterial colonies were found within the necrotic lesions (Figure 2C). Concomitantly, chorioallantoic membranes exhibited moderate to severe necrosis and suppurative inflammation of the epithelium (Figure 2D). In IHC analysis, large amounts of *C. jejuni* antigens were evident in trophoblastic epithelial cells lining the chorioallantoic membrane (Figure 2E and F); however, it was notable that under the IHC conditions, including the use of a single detection antibody, bacterial antigens were rarely detected in the placentomes and in the stroma of chorioallantoic membranes regardless of the ovine or bovine strains used and the severity of the lesions observed. These data indicated a possible tissue tropism of the abortion-associated *C. jejuni* in the chorioallantoic placenta of sheep.

**Figure 3:**
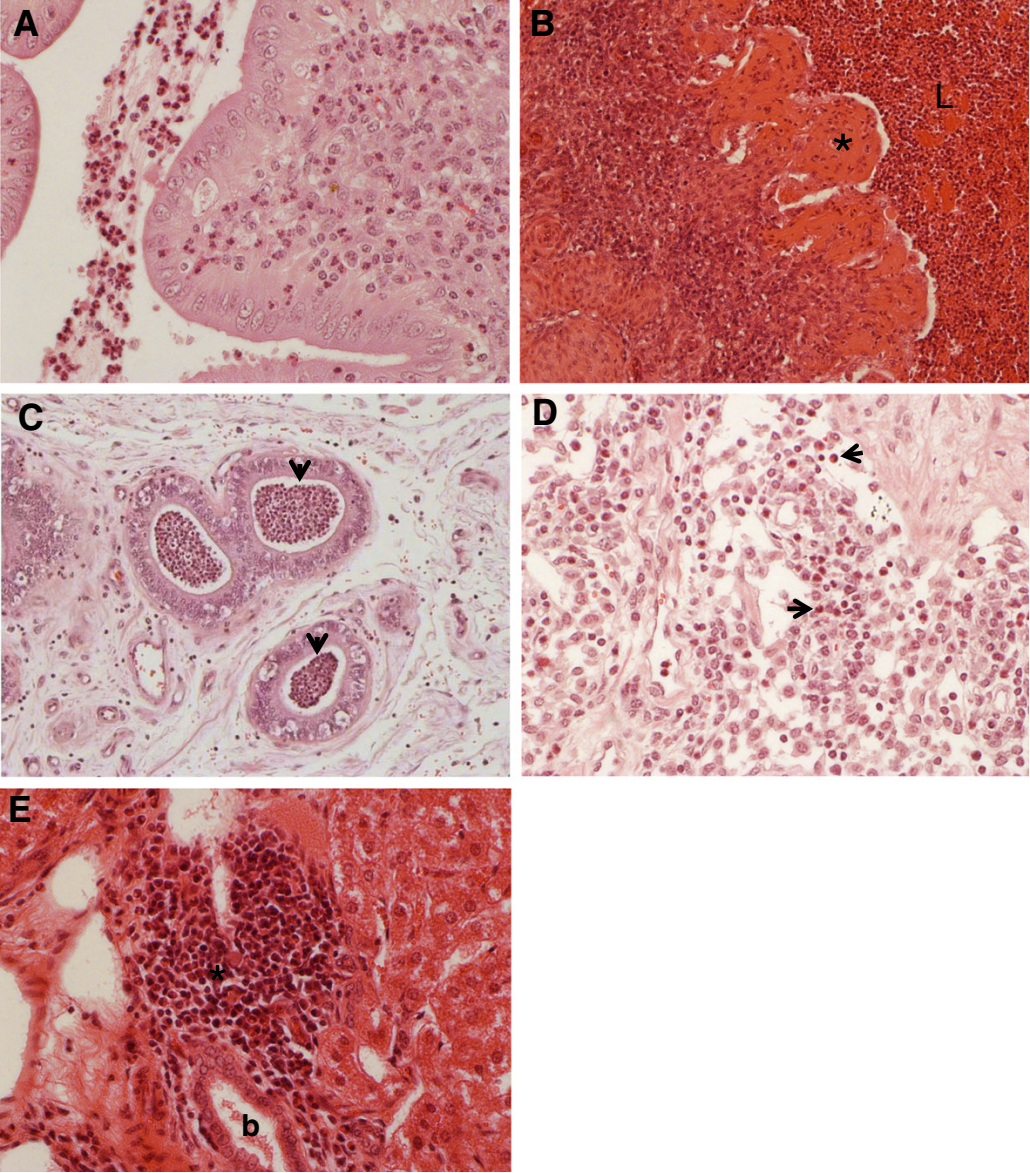
**Pathological findings in uterus and other organs of infected ewes. (A)** Uterus of an ewe experimentally infected with bovine *C. jejuni*, showing moderate suppurative endometritis. Original magnification × 400. **(B)** Uterus of an aborted ewe experimentally infected orally with ovine *C. jejuni*, showing severe necrotizing, suppurative endometritis, i.e. pyometra. Note severe necrosis of endometrium (asterisk) and a large accumulation of neutrophils and necrotic cells in the lumen of uterus (L). Original magnification, ×400. **(C)** Uterus of an ewe experimentally infected with bovine *C. jejuni*, showing suppurative endometritis characterized by accumulation of neutrophils in uterine glands (arrows). Original magnification, ×200. **(D)** Lymph node of an ewe experimentally infected with bovine *C. jejuni*, showing mild diffuse suppurative lymphadenitis (arrows). Original magnification, ×400. **(E)** Liver of an aborted ewe experimentally infected with ovine *C. jejuni*, showing focal suppurative periportal hepatitis. Note interstitial infiltration of polymorphonuclear neutrophils (asterisk) around the bile duct (b). Original magnification, ×400.

Moderate to severe endometritis observed in abortion associated *C. jejuni-*inoculated ewes was characterized by suppurative inflammation in the endometrium and in the lumen of uterus (Figure 3A), and occasional severe necrosis of endometrium accompanied by severe suppurative inflammation (Figure 3B). In G2 ewes infected i/v with the bovine abortion isolate, endometrial glands were filled with large numbers of neutrophils (Figure 3C). In addition, 2 ewes from G2 which were orally inoculated with the bovine isolate showed mild diffuse suppurative lymphadenitis (Figure 3D). Three ewes from G2 and G3 i/v inoculated with bovine or ovine strains, respectively, exhibited periportal suppurative hepatitis (Figure 3E). In G1 and the control group, no histological lesions were detected in cotyledons, endometrium, liver, spleen, and other major organs. Histological examination of intestinal tissues was not performed as these tissues underwent spontaneous autolysis.

**Figure 4:**
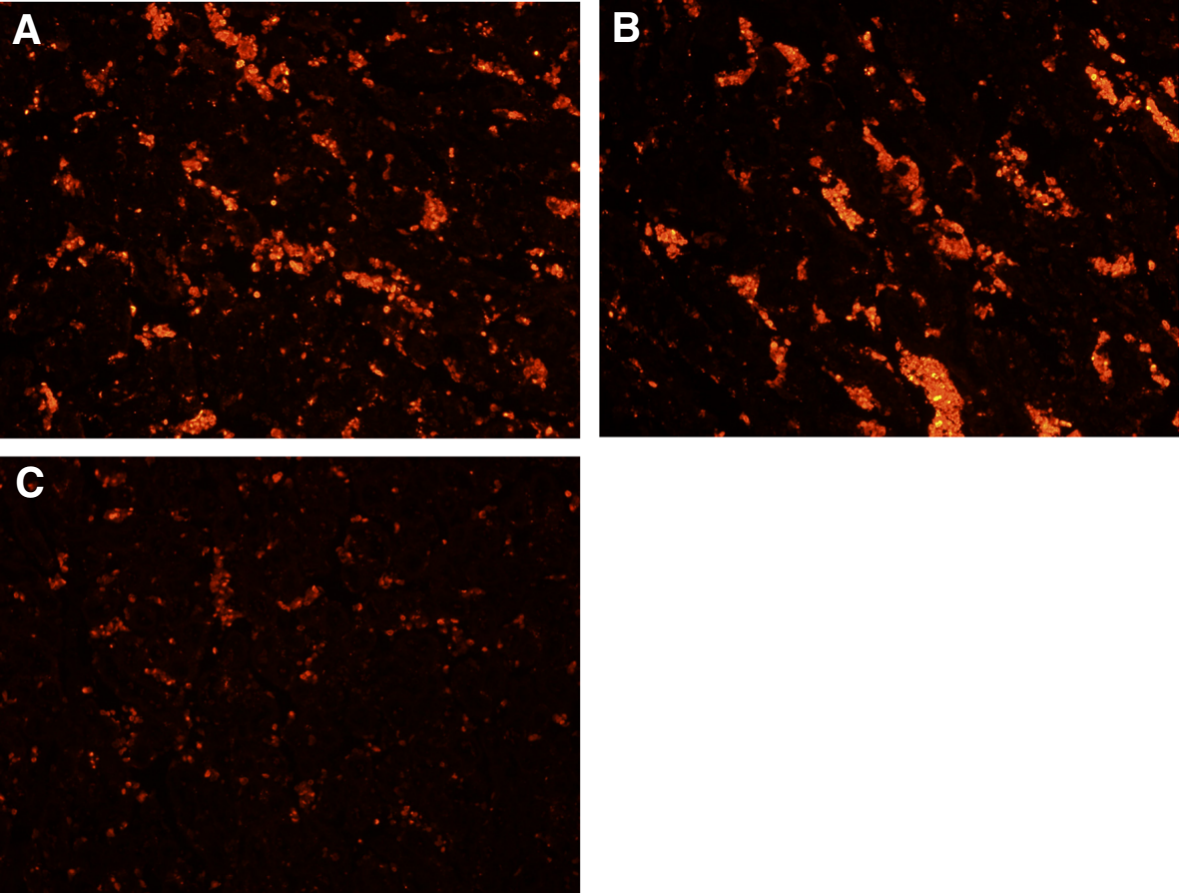
**Placentome stained by** ***in situ*** **TUNEL. (A)** After intravenous infection with bovine *C. jejuni* strain and **(B)** after oral infection with ovine *C. jejuni* strain, showing larger numbers of *in situ* TUNEL-positive cells (red staining), compared to **(C)** negative control. Original magnification, ×200.

Using the *in situ* TUNEL assay, relative to the negative control or 81–176 inoculated ewes, the placentomes of the ewes infected with bovine or ovine abortion *C. jejuni* strains showed *in situ* TUNEL-positive cells (red staining) (Figure 4). Most of TUNEL-positive cells appeared to be trophoblasts exfoliated from placental villi. The *in situ* TUNEL-positive signals were also detected in trophoblasts lining the chorioallantoic membrane of the infected ewes (data not shown).

### Bacteriological studies

*C. jejuni* was retrieved from feces of all the inoculated ewes at the time of necropsy except from two ewes (Table 1). *C. jejuni* was occasionally retrieved from several tissues of euthanized ewes including liver, spleen, uterus, placenta, blood, and small intestine in both G2 and G3 groups. However, *C. jejuni* was isolated by direct plating from placenta and uterus from the abortion and stillbirth cases from both the G2 and G3 with counts ranging from 4 × 10^5^ to 2 × 10^8^ CFU ml^-1^. *C. jejuni* was also retrieved in large numbers from lungs of one of the aborted ewes (G3-O-2) (Table 1). *C. jejuni* was also retrieved from small intestines of the four ewes in G1. Furthermore, *C. jejuni* was occasionally retrieved from lambs’ feces and fetuses’ meconium as well as small intestine and blood (Table 1).

### Host gene expression

Summary of transcriptome responses following *C. jejuni* administration is listed in Additional file 1: Table S1. The microarray generated transcripts were further mapped to available gene annotation in IPA. The number of IPA mapped genes was further compared across different treatment groups. Despite the poor annotation of the *Ovis aries* genome, BLAST searches against IPA knowledge database using human, rat, and mouse reference species allowed identification of a total of 51 mapped probes displaying expression changes greater or lesser than 2 folds between non-inoculated controls and 81–176, bovine, or ovine *C. jejuni* clone SA infected ewes from the complete set of differentially expressed genes (Table 2).

**Table 2:** **Variably expressed mapped genes in the uterine tissues of ewes in different groups**

**Gene description** ^**a**^	**Fold change**					
	**C-vs-G1**	**C-vs-G2**	**C-vs-G3**	**G1-vs-G2**	**G1-vs-G3**	**G2-vs-G3**
ASRGL1, Asparaginase like 1	3.14	2.09				
ATF4, Activating transcription factor 4 (tax-responsive enhancer element B67)	3.19	2.10				
BRS3, Bombesin-like receptor 3	3.07	2.38				
CAPN1, Calpain 1, (mu/I) large subunit			2.78	2.52		
CD28, CD28 molecule		-2.64				
CDKN2AIPNL, CDKN2A interacting protein N-terminal like			-3.15			
CHRD, Chordin		-1.90	-2.35	-2.32	-2.33	
CLN5, Ceroid-lipofuscinosis, neuronal 5	4.20			-2.17		
CYB561, Cytochrome b-561	-4.82		-4.81	2.80		
DOHH, Deoxyhypusine hydroxylase/monooxygenase	-5.27			3.64	5.85	
EEF1A1, Eukaryotic translation elongation factor 1 alpha 1	-7.51			7.17		
EGR1, Early growth response 1					2.10	2.00
EPAS1, Endothelial PAS domain protein 1	-5.61	-1.92		4.41	5.80	2.33
FGF7, Fibroblast growth factor 7	3.44	2.06	2.00			
FGFR2, Fibroblast growth factor receptor 2				-2.30		
GATA6, GATA binding protein 6		-1.91	-2.00			
GGCX, Gamma-glutamyl carboxylase			2.02	2.39	3.49	2.00
GNLY, Granulysin		2.31				
GNRH1, Gonadotropin-releasing hormone 1 (luteinizing-releasing hormone)		-2.18				
GRIA1, Glutamate receptor, ionotropic, AMPA 1			2.27			
HSPA5, Heat shock 70 kDa protein 5 (glucose-regulated protein, 78 kDa)				-2.09		
IFNAR2, Interferon (alpha, beta and omega) receptor 2				2.30		
IL5, Interleukin 5 (colony-stimulating factor, eosinophil)		2.47				
IL6, Interleukin 6 (interferon, beta 2)			2.02		1.95	2.21
IL12B, Interleukin 12B (natural killer cell stimulatory factor 2, cytotoxic lymphocyte maturation factor 2, p40)	3.06	2.14				
IL15, Interleukin 15	1.90	-2.06			3.60	2.00
INSL3, Insulin-like 3 (Leydig cell)		-2.06	-3.19			
ITGAL, Integrin, alpha L (antigen CD11A (p180), lymphocyte function- associated antigen 1; alpha polypeptide)		-1.99	-3.54			
ITGB2, Integrin, beta 2 (complement component 3 receptor 3 and 4 subunit)		-1.90				
MC1R, Melanocortin 1 receptor (alpha melanocyte stimulating hormone receptor)	-3.89	-2.82			3.19	2.32
MMP2, Matrix metallopeptidase 2 (gelatinase A, 72 kDa gelatinase, 72 kDa type IV collagenase)				2.10	2.40	
MMP9, Matrix metallopeptidase 9 (gelatinase B, 92 kDa gelatinase, 92 kDa type IV collagenase)				2.00		
MMP13, Matrix metallopeptidase 13 (collagenase 3)			2.00			
MYC, V-myc myelocytomatosis viral oncogene homolog	5.64	2.11		-2.66		
PCK1, Phosphoenolpyruvate carboxykinase 1 (soluble)		2.82				
PCNA, Proliferating cell nuclear antigen	-3.48			2.30	4.85	2.10
PGD, Phosphogluconate dehydrogenase	3.54	2.00				
PLIN5, Perilipin 5						2.04
PRL, Prolactin	-3.52			3.05	4.29	
PTGER3, Prostaglandin E receptor 3 (subtype EP3)			-2.39			
PTGER4, Prostaglandin E receptor 4 (subtype EP4)			-1.90		-2.00	-2.00
SLC4A4, Solute carrier family 4, sodium bicarbonate cotransporter, member 4	-3.55		-3.00	2.39		
SLC11A1, Solute carrier family 11			-2.36			
SLC25A5, Solute carrier family 25 (mitochondrial carrier; adenine nucleotide translocator), member 5	-4.06				3.15	
SCNN1A, Sodium channel, non-voltage-gated 1 alpha subunit					2.89	2.09
SLPI, Secretory leukocyte peptidase inhibitor				2.45	3.71	
ST3GAL4, ST3 beta-galactoside alpha-2,3-sialyltransferase 4	3.70			-2.58		
TFF3, Trefoil factor 3 (intestinal)				-2.21		
TRAF3, TNF receptor-associated factor 3				1.94	2.00	
UGT1A9, UDP Glucuronosyltransferase 1 family, polypeptide A9			-5.24	2.49		
ZGPAT, Zinc finger, CCCH-type with G patch domain	2.99					

^a^Gene description is based on the Ingenuity Pathway Analysis (IPA) database. C, Control; G1, infected with 81–176; G2, infected with Bovine abortion isolate; G3, infected with Ovine abortion-II isolate. + Upregualted; - Downregulated.

IPA was used for gene ontology (GO) analysis. Datasets from a total of 6 groups were analyzed for GO; (control) cont-vs- G1 (81–176 strain), cont-vs-G2 (bovine strain), and cont-vs-G3 (ovine strain), G1-vs-G2, G1 vs G3 and G2 vs G3. Of the 51 genes analyzed, 14 (~27%) of up- and/or -down regulated genes were common among different datasets (Table 2), 23 (45%) were up-regulated only, and 14 (~27%) were down-regulated across all groups compared. The genes encoding extracellular marker proteins and other proteins involved in immune responses, cellular apoptosis and necrosis, embryonic development, cellular growth, proliferation and differentiation, and organismal injury and abnormalities were also differentially expressed among the different comparisons (Table 2). Of particular interest, several genes evidently showed down shift expression in animals from G2 and G3 compared to either G1 (infected with 81–176 *C. jejuni*) or the control non-inoculated group (Table 2). For example, the gene encoding GATA binding protein 6 (GATA6) was down-regulated by approximately 2 folds in G2 and G3 as compared to the control non-inoculated group. Similarly, gene encoding insulin-like 3 (Leydig cell; INSL3) was downregulated by approximately 2 and 3 folds in G2 and G3, respectively, compared to the negative control group. The gene encoding chordin (CHRD) was down-regulated by approximately 2 and 2.4 folds in G2 and G3, respectively. However, several genes were up-regulated particularly in aborted ewes from G2 and G3 compared to either G1 or the control non-inoculated group. For example, the gene encoding matrix metallopeptidase 2 (MMP2) was up-regulated by around 2.1 and 2.4 folds in G2 and G3, respectively. The gene encoding tumor necrosis factor receptor-associated factor 3 (TRAF3) was up-regulated by around 2 folds in both G2 and G3 as compared to G1. The gene encoding gamma-glutamyl carboxylase (GGCX) was up-regulated by 2.4 and 3.5 in G2 and G3, respectively (Table 2).

Of particular interest, expression patterns of several genes were found only in G3 group ewes. Interleukin 6 (IL-6) was up-regulated approximately 2.0 fold in the G3 compared to G1, G2, and control non-inoculated group ewes. IL-15 was up-regulated in G3 by 3.6 and 2 folds compared to G1 and G2, respectively. The gene encoding early growth response protein (EGR1) was up-regulated by around 2 folds in G3 as compared to G1 and G2. Whereas, the gene encoding prostaglandin E receptor 4 (PTGER4) was down-regulated by around 2 folds in G3, as compared to G1, G2, and control group ewes (Table 2). In general, the data generated from the microarray analysis, mainly the differential expression of certain genes, gave further insights and supported the clinical and histopathological observations.

## Discussion

Pathogenesis of *C. jejuni* clone SA induced abortion is not well understood. Recently, it was shown that the *C. jejuni* clone SA can effectively induce abortion in pregnant guinea pigs [9]. However, no studies have characterized the virulence of these strains in a natural host (sheep). In this study, under experimental conditions, abortion-associated *C. jejuni* belonging to clone SA seemed to effectively induce abortion or stillbirth in pregnant sheep when high doses of inoculum (10^9^ CFU in 1.5 ml of SS for i/v) were used. This corroborated a previous study [8] and was further confirmed in our preliminary trials where no abortion was observed in pregnant ewes infected with lower doses of *C. jejuni* (10^6^ CFU in 1.5 ml of SS for i/v), although several significant clinical signs such as uterine prolapse and retained placenta were noted (data not shown). Additionally, based on our preliminary trials, the differences in the pregnancy stages of ewes might result in disparity in the induction of abortion. However, when using the higher dose of *C. jejuni* and ewes with synchronized pregnancies, typical abortion occurred in one (G3-O-2) of the ewes that were orally inoculated with the ovine isolate from G3 at PID 15 (Figure 1A). Stillbirths were also observed in 2 cases (G2-V-1 and G2-V-2) from G2 and one case (G3-O-1) from G3, which were inoculated i/v and orally with bovine and ovine isolates, respectively (Table 1). Furthermore, one ewe (G3-V-1) infected i/v, died 20 h post-inoculation and in the other ewe (G3-V-2) no *C. jejuni* was isolated from any tissues. However, at postmortem G3-V-2 was found to be heavily infested with tapeworm, which might have affected *C. jejuni* colonization. The cause of sudden death might be due to acute septicemia and uterine sepsis or due to the septic shock which can be caused by the systemic inflammatory response to the bacteremia of Gram negative bacteria [4,15].

Macroscopic observations in aborted ewes or fetuses in this study were mostly in agreement with previous findings [5,8]. Gross lesions that mostly consisted of hemorrhagic fibrinosuppurative placentitis and endometritis were evident in the majority of ewes infected with the abortion-associated *C. jejuni* (Figure 1B). In addition, petechial hemorrhages were seen on the liver and spleen of i/v inoculated ewes from both groups (G2 and G3). In the guinea pig model, rather than hemorrhagic lesions, multiple round and white foci of variable sizes were identified on liver in 90% of pregnant guinea pigs infected with ovine abortion inducing *C. jejuni* strain [9].

Pregnant ewes i/v or orally infected with the abortion-associated *C. jejuni* exhibited histopathological changes in the placental and uterine tissues. Based on this observation, it is presumed that these strains might employ bacteremia to establish their infection in the reproductive organs. Using IHC, bacterial antigens were mainly detected in trophoblasts lining the chorioallontoic membrane, usually undergoing necrosis (Figure 2E and F). Placentomes also exhibited severe suppurative inflammatory and necrotic lesions possibly caused by *C. jejuni*, which was frequently observed within the lesions (Figure 2C and D). Similar necrotic lesions with numerous large *C. jejuni* aggregates (colonies) in the placental villi and adjacent stroma were previously identified in inoculated ewes [8]. These data indicate a possible cellular tropism of abortion-associated *C. jejuni* in the placental epithelial cells possibly transported via the bloodstream. Consistent with suppurative inflammatory lesions in other major organs, such as uterus, liver, spleen, and lymph node, the bacteriological examination revealed *C. jejuni* in multiple tissues, including liver, spleen, uterus, placenta, blood, small intestine of infected ewes, as well as from offspring’s feces, fetuses’ meconium, small intestine and blood as shown in Table 1. Based on the data, the abortion-associated *C. jejuni* might be able to induce systemic infection, usually accompanied by intestinal and reproductive infection. In the pregnant guinea pig model, large numbers of *C. jejuni* were mainly retrieved from placental and uterine tissues, while small to moderate numbers of bacteria were recovered from feces and blood as well as from fetal liver and lung samples [9]. Similarly, high counts of *C. jejuni* were detected in uterine and placental tissues and from stomach content, bile, and feces of ewes infected with ovine abortion *C. jejuni* as well as from aborted fetuses [8].

To acquire more insights into the pathogenic mechanisms of abortion-associated *C. jejuni*, host gene expression profiles in uterine tissues from aborted ewes were determined using microarray analysis. The genes encoding GATA binding protein 6 (GATA6) and chordin (CHRD) that account for embryonic development [22,23] and dorsalization of early vertebrate embryonic tissues [24], respectively, were down-regulated in G2 and G3 vs. the control non-inoculated group or the G1 infected with 81–176 (Table 2). In addition, insulin-like 3 (Leydig cell; INSL3) that is essential for the embryonic development [25] was down-regulated in G2 and G3 vs. the control group. These data suggest that the impairment of embryonic development caused by abortion-associated *C. jejuni* infection might be in part responsible for abortion of fetuses identified in the G3.

In the G3 infected with ovine *C. jejuni*, the gene encoding EGR1 which stimulates apoptosis and inflammatory response [26] was up-regulated compared to G1 and G2. Concomitantly, there was an increase in the gene expression of a pro-inflammatory cytokine, IL-6, in G3 compared to G1 and G2 (Table 2). Elevated levels of IL-6 in the placenta, amniotic cells, and endometrium have been demonstrated in pregnancies complicated by preterm premature rupture of the membranes, intrauterine infection, and prematurity [27,28]. Consistent with the increased levels of prostaglandin receptor EP4 (Ptger4) in uterine tissue of IL6-/- mice compared with wild type mice [29], down-regulation of Ptger4 gene was significantly observed in the G3 showing increased IL-6. In addition to increased expression of IL-6 gene, up-regulation in the gene expression of TNF receptor associated factor 3 (TRAF3) was detected in the G3 vs. G1 and G2. The TRAF3 plays a role in the activation of the immune response [30]. Increased pro-inflammatory responses detected in the lesions in this study might be in part implicated in increased apoptotic death of infected placental cells.

The IL-15 plays a regulatory role in mediating the inflammatory environment [31,32]. Of interest, this cytokine might be associated with abortion [33,34]. Previous studies showed that up-regulation of IL-15 gene in trophoblasts contributed to recurrent abortions, suggesting that this cytokine can be a marker for pregnancy failure [33,34]. Similar to increased expression of IL-6 gene stated earlier, up-regulation of IL-15 gene expression was detected in the G3 and G2 vs. the negative control. The activated immunological status during pregnancy after inoculation with the abortion-associated *C. jejuni* might be implicated in the related abortive pathogenesis. The increased apoptotic cell death in placental tissues particularly from aborted ewes in G2 and G3 vs. the G1 or control group (Figure 4) might be related to the increased responses of pro-inflammatory cytokine, IL-6, and IL-15 as described previously [35-37].

Both the ovine and bovine abortion isolates possess identical sequence types (ST-8) (Additional file 1: Figure S1). ST-8 has been previously recorded in different hosts, including humans [5,13], thus the abortion isolates may have zoonotic potential warranting further investigation of their implications to public health. Although there are no reports on associating clone SA to human abortions, the highly abortificient nature and the presence of large number of bacteria in aborted materials as well as in fetal tissues [9] may pose a potential environmental risk for zoonotic infections. The latter is also indicated by existing evidence that suggest that the clone SA is associated with human gastroenteritis [5,7]. Both ovine and bovine abortion associated *C. jejuni* in this study were capable of invading the human intestinal epithelial cells (INT407). Specifically, ovine abortion-II was as invasive in INT407 as the hyper-invasive strain *C. jejuni* 81–176 (Additional file 1: Figure S2) and showed significantly higher intracellular survival than that of the NCTC11168 (*P* < 0.05), a poorly invasive strain [38]. These findings further highlight the potential public health impact of the abortion associated *C. jejuni* strains.

## Conclusions

The results of this study revealed that bovine and ovine *C. jejuni* abortion isolates have the potential to induce necrotizing suppurative placentitis and endometritis, thereby causing abortion or stillbirth in pregnant sheep. Necrotic lesions and suppurative inflammation in the placenta and endometrium coincided with increased expression of the genes related to cellular necrosis and pro-inflammatory responses accompanied by decreased expression of the genes that account for embryonic development. The distinct genomic contents and transcriptome responses of clone SA may facilitate its systemic spread and subsequent localization in the sheep uterus leading to abortion. However, further studies are needed to define the bacterial factors contributing to clone SA’s unique virulence and pathogenesis in sheep. Although a relatively limited number of animals was used in this study, the results show that, sheep as an animal model, can provide useful and novel insights into the pathogenesis and the host responses triggered by bovine and ovine abortion-associated *C. jejuni* clone SA.

## Additional file

Additional file 1: Table S1 Summary of transcriptome responses. **Figure S1.** *Sma*I Macro Restriction Profiles, Sequence Types and antimicrobial resistance profiles of ovine and bovine *C.jejuni* abortion-associated isolates. **Figure S2.** Invasion and intracellular survival of ovine and bovine *C. jejuni* isolates in INT407 cells.
