# Supplementary material for: Insights into potential pathogenesis mechanisms associated with Campylobacter jejuni-induced abortion in ewes
Source: BMC Vet Res. 2014 Nov 25;10:274. doi: 10.1186/s12917-014-0274-8 (PMC4262353; doi:10.1186/s12917-014-0274-8)
Supplement: Additional file 1: Table S1 — Summary of transcriptome responses. Figure S1. SmaI Macro Restriction Profiles, Sequence Types and antimicrobial resistance profiles of ovine and bovine C.jejuni abortion-associated isolates. Figure S2. Invasion and intracellular survival of ovine and bovine C. jejuni isolates in INT407 cells. [file 12917_2014_274_MOESM1_ESM.pdf]

Table S1. Summary of transcriptome responses.

Groups	Total [a]	Up-regulated	Down-regulated
Control vs G1	791	334	457
Control vs G2	227	95	132
Control vs G3	293	54	239
G1 vs G2	370	284	86
G1 vs G3	723	386	337
G2 vs G3	301	92	209

[a]Total number of differentially expressed probes. Differential expression of probes were determined using a fold change cutoff ± ≥ or ≤ 2

Supplemental Figure Legends

Fig. S1. *Sma*I Macro Restriction Profiles, Sequence Types and antimicrobial resistance profiles of ovine and bovine *C.jejuni* abortion-associated isolates. Similarity analysis was performed using the Dice coefficient, and clustering was performed by the unweighted pair-group method with arithmetic averages UPGMA (optimization, 1% and position tolerance, 1.5%) (Sanad et al., 2011; Ribot et al., 2001). Numbers on bootstraps represent Cophenetic correlations. Black boxes indicate resistance to different antimicrobials tested as described previously (Sanad et al., 2011; Sanad et al., 2013). Antimicrobials; azithromycin (AZ) (Breakpoint for a resistant *Campylobacter* isolate: ≥8 µg ml^{-1}); ciprofloxacin (CI) (≥4 µg ml^{-1}); erythromycin (ER) (≥32 µg ml^{-1}); gentamicin (GE) (≥8 µg ml^{-1}); tetracycline (TE) (≥16 µg ml^{-1}); florfenicol (FF) (≥8 µg ml^{-1}); nalidixic acid (NA) (≥64 µg ml^{-1}); telithromycin (TL) (≥8 µg ml^{-1}); and clindamycin (CL) (≥8 µg ml^{-1}). Minimal inhibitory concentrations (MIC) were determined according to the Clinical and Laboratory Standards Institute (CLSI 2006). Multilocus sequence typing (MLST) was conducted as described previously (Sanad et al., 2011; Dingle et al., 2001).

Fig. S2: Invasion and intracellular survival of ovine and bovine *C. jejuni* isolates in INT407 cells. Invasion and survival assays were performed as described previously (Sanad et al., 2011; Konkel et al., 1992; Prasad et al., 1996). **A.** CFU ml^{-1} representing the number of the internalized bacteria which could be retrieved after treatment of cells with gentamicin. **B.** Intracellular survival of *C. jejuni* isolates in INT 407cells. CFU ml^{-1} representing the numbers of internalized bacteria retrieved after 24 h of incubation. The INT407 were infected with 1:100 MOI of *C. jejuni* strains. *C. jejuni* 81–176 and NCTC11168 were used as controls. The detection limit of the assay is represented by the dashed line. Each bar represents the mean ± SE of three independent experiments performed in duplicate for each sample (P<0.01).

Fig. S1

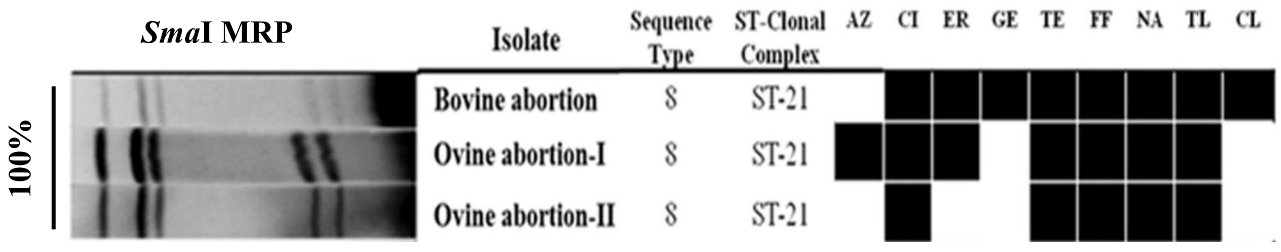
SmaI MRP
100%
Isolate
Sequence Type
ST-Clonal Complex
AZ
CI
ER
GE
TE
FF
NA
TL
CL
Bovine abortion
8
ST-21
Ovine abortion-I
8
ST-21
Ovine abortion-II
8
ST-21

Fig. S2:

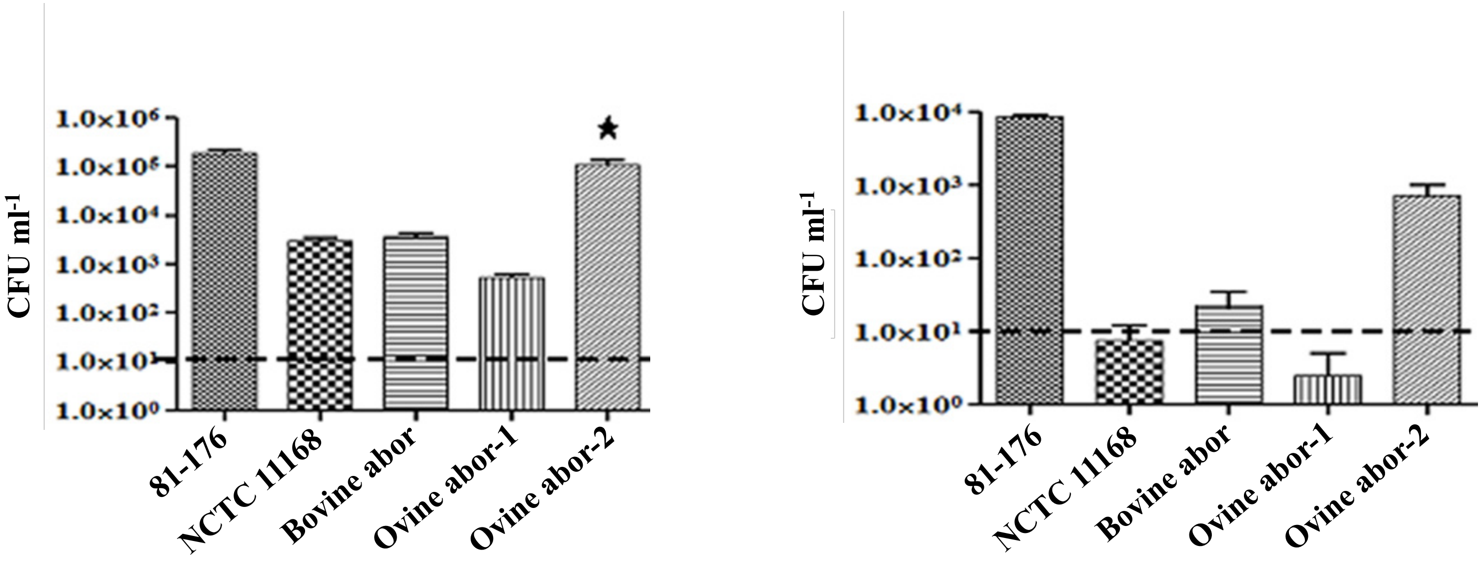
CFU ml-1
1.0×10⁶
1.0×10⁵
1.0×10⁴
1.0×10³
1.0×10²
1.0×10¹
1.0×10⁰
81-176
NCTC 11168
Bovine abor
Ovine abor-1
Ovine abor-2
CFU ml-1
1.0×10⁴
1.0×10³
1.0×10²
1.0×10¹
1.0×10⁰
81-176
NCTC 11168
Bovine abor
Ovine abor-1
Ovine abor-2

References

Clinical and Laboratory Standards Institute (CLSI), 2006: Performance standards for antimicrobial disk susceptibility tests, approved standard, 9th ed. Clinical and Laboratory Standards document M2-A9. Clinical and Laboratory Standards Institute, Wayne, PA.

Dingle, K. E., F. M. Colles, D. R. A. Wareing, , Ure R., A. J. Fox, F. E. Bolton, H. J. Bootsma, R. J. L. Willems, R. Urwin, and M. C. J. Maiden, 2001: Multilocus sequence typing system for *Campylobacter jejuni*. *J. Clin. Microbiol*. 39, 14–23.

Konkel, M. E,. and W. Cieplak Jr, 1992: Altered synthetic response of *Campylobacter jejuni* to cocultivation with human epithelial cells is associated with enhanced internalization. *Infect. Immun*. 60, 4945-4949.

Prasad, K. N., T. N. Dhole, and A. Ayyagari, 1996: Adherence, invasion and cytotoxin assay of *Campylobacter jejuni* in HeLa and HEp-2 cells. *J. Diarrhoeal Dis. Res*. 14, 255–259.

Ribot, E. R., C. Fitzgerald, K. Kubota, B. Swaminathan, and D. Barrett, 2001: Rapid pulsed-field gel electrophoresis protocol for subtyping of *Campylobacter jejuni*. *J. Clin. Microbiol*. 39, 1889–1894.

Sanad, Y. M., I. I. Kassem, M. Abley, W. Gebreyes, J. T. LeJeune, and G. Rajashekara, 2011: Genotypic and phenotypic properties of cattle-associated *Campylobacter* and their implications to public health in the USA. *PLoS ONE*. 6, e25778.

Sanad, Y. M., G. Closs Jr, A. Kumar, J. T. LeJeune, G. and Rajashekara, 2013: Molecular Epidemiology and Public Health Relevance of *Campylobacter* Isolated from Dairy Cattle and European Starlings in Ohio, USA. *Foodborne Path. Dis*. 10, 229-236.
